# Influence of Angiotensin-Converting-Enzyme Gene Polymorphism on Echocardiographic Data of Patients with Ischemic Heart Failure

**DOI:** 10.5935/abc.20160145

**Published:** 2016-11

**Authors:** Gustavo Salgado Duque, Dayse Aparecida da Silva, Felipe Neves de Albuquerque, Roberta Siuffo Schneider, Alinne Gimenez, Roberto Pozzan, Ricardo Mourilhe Rocha, Denilson Campos de Albuquerque

**Affiliations:** 1 Hospital Universitário Pedro Ernesto - Universidade Estadual do Rio de Janeiro (UERJ); RJ - Brazil; 2 Laboratório de Diagnósticos por DNA do Instituto de Biologia da Universidade do Estado do Rio de Janeiro, RJ - Brazil

**Keywords:** Heart Failure, Polymorphism, Genetic, Angiotensin-Converting Enzyme Inhibitors, Echocardiography / methods

## Abstract

**Background:**

Association between angiotensin-converting-enzyme (ACE) gene polymorphisms and different clinical and echocardiographic outcomes has been described in patients with heart failure (HF) and coronary artery disease. Studying the genetic profile of the local population with both diseases is necessary to assess the occurrence of that association.

**Objectives:**

To assess the frequency of ACE gene polymorphisms in patients with ischemic HF in a Rio de Janeiro population, as well as its association with echocardiographic findings.

**Methods:**

Genetic assessment of I/D ACE polymorphism in association with clinical, laboratory and echocardiographic analysis of 99 patients.

**Results:**

The allele frequency was: 53 I alleles, and 145 D alleles. Genotype frequencies were: 49.5% DD; 47.48% DI; 3.02% II. Drug treatment was optimized: 98% on beta-blockers, and 84.8% on ACE inhibitors or angiotensin-receptor blocker. Echocardiographic findings: difference between left ventricular diastolic diameters (ΔLVDD) during follow-up: 2.98±8.94 (DD) vs. 0.68±8.12 (DI) vs. -11.0±7.00 (II), p=0.018; worsening during follow-up of the LV systolic diameter (LVSD): 65.3% DD vs. 19.0% DI vs. 0.0% II, p=0.01; of the LV diastolic diameter (LVDD): 65.3% DD vs. 46.8% DI vs. 0.0% II, p=0.03; and of the LV ejection fraction (LVEF): 67.3% DD vs. 40.4% DI vs. 33.3% II, p=0.024. Correlated with D allele: ΔLVEF, ΔLVSD, ΔLVDD.

**Conclusions:**

More DD genotype patients had worsening of the LVEF, LVSD and LVDD, followed by DI genotype patients, while II genotype patients had the best outcome. The same pattern was observed for ΔLVDD.

## Introduction

Heart failure is a complex syndrome, and there is strong evidence that gene polymorphisms play an important role in its pathophysiology and progression.^1,2^ In addition, neuro-hormonal activation has a role in heart failure course. Angiotensin-converting-enzyme (ACE), a key player in the renin-angiotensin-aldosterone system, is essential to heart function regulation.^3,4^

Angiotensin-converting-enzyme gene polymorphisms (ACEGP) have been associated with heart failure prognosis, and several studies have shown the association of D allele and DD genotype with worse echocardiographic outcomes in patients with systolic dysfunction.^5,6^

The DD genotype is associated with higher frequency of acute myocardial infarction in several populations, in addition to major ischemic defects after occlusion of a coronary artery.^7,8^

Coronary artery disease (CAD) is a common cause of heart failure,^9^ and, similarly to the presence of the D allele and DD genotype, is associated with both CAD and heart failure independently.^5,10^ Thus, we decided to study the frequency of ACEGP in a population of patients with CAD and heart failure, assessing their echocardiographic findings, and comparing them in the different genotype groups.

## Methods

Observational, retrospective cohort of 3 years and 4 months, with data collected from the medical records of patients of a university-affiliated hospital, in addition to genetic analysis at the same university.

This study assessed 101 patients, 99 of whom completed the genotyping process for ACE gene alleles, constituting this study's sample. The alleles were determined at the time of patients' inclusion in the study, their clinical follow-up being then retrospectively assessed.

The patients were assessed by a multidisciplinary team, their guidance and treatment following the Brazilian Society of Cardiology guidelines. Data were collected during visits to the outpatient clinic by doctors participating in the study, and were reviewed by the main author of the study.

The inclusion criteria were as follows: age over 18 years; heart failure diagnosis according to the Framingham criteria; left ventricular ejection fraction (LVEF) <50% on echocardiography, assessed with the Simpson's method at any time of clinical follow-up; CAD demonstrated on coronary angiography with evidence of significant obstructive disease (≥ 75%)^11^ or previous acute myocardial infarction or previous percutaneous coronary angioplasty or surgical myocardial revascularization. The exclusion criteria were as follows: unavailable or inappropriate medical records; non-ischemic etiology of heart failure; and loss to follow-up by the end of the study.

This study was approved by the Ethics Committee of the University, being included in the Brazilian system of Ethics in Research. All patients provided written informed consent before the beginning of the study, which abided by the principles of the Declaration of Helsinki.

The procedures of data analysis and collection from the medical records were blind to the researchers. The genotype was known only at the end of the review of the medical record; therefore, no physician knew that information at the time of the medical visits.

Skin color was observed by the physician, the individuals being classified as white, black, mixed or other (yellow/Asian).

### Echocardiographic variables

All patients underwent at least two echocardiographic assessments at different times, undergoing new tests at the clinical discretion of the medical team. Data of the first echocardiography and of another conducted at the end of the follow-up were collected, in two device models, GE Vivid 3 and HD7 Philips, with a 2.75-MHz transducer, the test being performed by a physician blinded to the patients' genotypes.

The following echocardiographic data were assessed: LVEF (Simpson's method); left ventricular systolic and diastolic diameters (LVSD and LVDD, respectively). The methodology to measure echocardiographically the ventricular diameters and muscle thickness followed the rules of the American Society of Echocardiography.

Echocardiographic outcomes were assessed by calculating the differences between the final and initial values of the parameters measured (LVEF, LVSD and LVDD) as follows: variation of the left ventricular ejection fraction (ΔLVEF), variation of the LVSD (ΔLVSD), and variation of the LVDD (ΔLVDD). In addition, objective improvement or worsening of those parameters during follow-up was assessed, with the creation of the following variables: FΔLVEF, for LVEF improvement or worsening during follow-up; FΔLVSD and FΔLVDD, for improvement or worsening of LVSD and LVDD, respectively, during follow-up.

### Genetic analysis

Blood samples were collected and stored at 5-15ºC for genetic analysis with DNA extraction, according to the salting-out method, genotyping with polymerase chain reaction, and later classification as DD, DI or II genotypes.

### Statistical analysis

All data obtained were analyzed with an IBM PC computer by using the SPSS for Windows statistical program, version 17.0 of 2008. The following tests were used: Tukey, chi-square (χ^2^), analysis of variance (F) and Pearson correlation. The statistical significance level adopted was 5%. Categorical variables were presented as absolute values and their respective percentages. Continuous variables were presented as mean ± standard deviation. To assess the distribution of the variables studied, skewness analysis was used. Gene and haplotype frequencies were tested for Hardy-Weinberg equilibrium, using ARLEQUIN software, version 2000.

### Weight of D allele

In addition to categorizing ACE genotypes into three groups (DD, DI and II) and assessing their relationship with the other variables, an analysis model was elaborated to test the isolated impact of each D allele on echocardiographic findings. Thus, a mathematical model was created to simulate the behavior of the ACE gene codominance, in which each copy of the D allele was assigned weight 1 in the analysis, so that the genotypes had the following weights: 0 (II genotype), 1 (DI genotype) and 2 (DD genotype), depending on the number of D alleles. Therefore, a categorical variable of ACEGP was transformed into a numerical variable (0, 1, 2) to simulate the weight of each copy of the D allele in the echocardiographic findings.

## Results

### Genetic profile of the sample

Regarding the allele frequency, I alleles occurred 53 times, while D alleles, 145 times. Genotype frequencies were 3.02% II, 47.48% DI and 49.5% DD. The genetic profile was tested and showed no deviation from the Hardy-Weinberg equilibrium.

### Characteristics of the population

Mean age was 65.4±11.4 years, with a wide range (36 years - 94 years). The distribution of skin color was as follows: white, 69.7%; mixed, 16.2%; black, 14.1%. There were no Asians. There were more males (73 men and 26 women) in the population and in the groups with D alleles, but not in the II group. There were more white individuals in all groups, with lower evidence in the DD group, with no statistically significant difference (Table 1). Drug treatment was assessed, and most patients were on ACE inhibitors and beta-blockers. There was no statistically significant variation between the gene groups assessed (Table 1).

**Table 1:** Clinical characteristics, tests and drug treatment of the population

Variable	Total (n=99)	DD (n=49)	DI (n=47)	II (n=3)	Statistical test	p
Age	65.40±11.42	65.38±12.41	65.34±10.27	66.64±16.28	F= 0.018	0.982
Male sex	73 (73.7%)	37 (75.5%)	35 (74.5%)	1 (33.3%)	Χ^2^ = 2.621	0.270
Female sex	26 (26.3%)	12 (24.5%)	12 (25.5%)	2 (66.7%)		
White color	69 (69.7%)	28 (57.1%)	38 (80.9%)	3 (100%)	Χ^2^ = 8.525	0.074
Non-white/non-black	16 (16.2%)	10 (20.4%)	6 (12.8%)	0 (0%)		
Black color	14 (14.1%)	11 (22.4%)	3 (6.4%)	0 (0%)		
T. diagn (months)	108.10±86.50	107.84±90.76	102.07±79.23	206.81±95.28	F= 2.115	0.126
Follow-up (months)	54.95±43.57	56.43±45.74	53.43±41.62	54.70±53.53	F= 0.056	0.946
Weight (Kg)	74.437±15.23	72.62 ±17.94	76.16±12.14	77.13±10.33	F= 0.694	0.502
Height (m)	1.64±0.87	1.63±0.93	1.65 ±0.80	1.60±0.93	F= 0.795	0.455
BMI (kg/m^2^)	27.66±4.83	27.06±5.11	28.13 ±4.52	30.09±4.9	F= 0.976	0.381
AC (cm)	96.14±11.71	94.48±12.52	97.39±10.87	102.5±9.99	F= 1.190	0.309
SAH	79 (79.8%)	37 (75.5%)	39 (83.0%)	3 (100%)	Χ^2^ = 1.613	0.446
DM	32 (32.3%)	15 (30.6%)	15 (31.9%)	2 (66.7%)	Χ^2^ = 1.687	0.430
Smoking currently	9 (9.1%)	6 (12.2%)	2 (4.3%)	1 (33.3%)	Χ^2^ = 5.132	0.274
Ex-smoker	54 (54.5%)	26 (53.1%)	26 (53.3%)	2 (66.7%)		
Never smoked	36 (36.4%)	17 (34.7%)	19 (40.4%)	0 (0%)		
Alcoholism currently	12 (12.1%)	6 (12.2%)	6 (12.8%)	0 (0%)	Χ^2^ = 5.931	0.204
Ex-alcoholic	17 (17.2%)	9 (18.4%)	6 (12.8%)	2 (66.7%)		
Dyslipidemia	75 (75.8%)	38 (77.6%)	34 (72.3%)	3 (100%)	Χ^2^ = 1.345	0.511
FH HF	8 (8.1%)	3 (6.1%)	5 (10.6%)	0 (0%)	Χ^2^ = 0.931	0.628
FH CAD	46 (46.5%)	23 (46.9%)	23 (48.9%)	0 (0%)	Χ^2^ = 2.724	0.256
SBP1 (mmHg)	126.27 ± 20.52	127.35 ± 20.22	124.64±20.86	134.33±25.03	F= 0.443	0.644
DBP1 (mmHg)	75.46±12.93	75.98±12.23	74.60±13.47	80.67±19.01	F= 0.383	0.683
HR1 (bpm)	73.06±14.85	72.86±14.42	72.02±14.73	92.67±14.01	F= 2.839	0.063
SBP2 (mmHg)	116.02±16.49	117.31±15.76	113.68±15.15	131.67±39.31	F= 2.014	0.139
DBP 2 (mmHg)	71.79±10.68	72.76±10.49	70.21±10.49	80.67±14.74	F= 1.776	0.175
HR2 (bpm)	70.27±11.80	71.57±11.52	69.04±12.32	69.33±8.51	F= 0.529	0.591
ΔSBP (mmHg)	-10.25±20.45	-10.04±21.11	-10.96±19.75	-2.67±27.03	F= 0.233	0.792
ΔDBP (mmHg)	-3.68±14.30	-3.22±13.75	-4.38±14.39	0±26	F= 0.178	0.837
ΔHR (bpm)	-2.79±15.67	-1.35±14.58	-2.98±15.80	-23.33±22.50	F= 2.897	0.06
Hb (g/dL)	13.72 ± 1.71	13.32 ± 1.90	14.12±1.39	14.00±2.17	F= 2.790	0.066
UA (mg/dL)	6.42±2.28	6.72±2.02	6.01±2.52	7.83±0.681	F= 1.797	0.171
TC (mg/dL)	178.19±52.12	176.27±56.81	183.64±46.22	124.33±37.54	F= 1.927	0.151
Na (mEq/L)	138.66±3.79	138.37±3.94	139.02±3.47	137.67±7.10	F= 0.457	0.635
Cr (mg/dL)	1.28±0.99	1.45±1.31	1.10±0.48	1.38±0.45	F= 1.592	0.209
CrCl (ml/min)	70.30±31.29	66.68±36.19	75.17±25.20	53.25±24.42	F= 1.352	0.264
QRS>120ms	14 (14.1%)	6 (12.2%)	7 (14.9%)	6 (12.2%)	Χ^2^=1.077	0.584
LBBB	21 (21.2%)	10 (20.4%)	10 (20.4%)	1 (33.3%)	Χ^2^= 0.283	0.868
BB	97 (98.0%)	49 (100%)	45 (95.7%)	3 (100%)	Χ^2^=2.258	0.323
BB target	65.49%±3.9%	60.59%±5.3%	70.63%±5.8%	66.67%±16.7%	F= 0.825	0.441
ACEI	47 (47.5%)	20 (40.8%)	24 (51.1%)	3 (100%)	Χ^2^=4.433	0.109
ACEI target	46.35%%±4.5%	46.35%±7.4%	42.06%±5.4%	75%±25%	F= 1.551	0.223
ARB	37 (37.4%)	20 (40.8%)	17 (36.2%)	0 (0%)	Χ^2^=2.068	0.356
Spiro	37 (37.4%)	18 (36.7%)	19 (40.4%)	0 (0%)	Χ^2^=1.986	0.370
Digitalis	19 (19.2%)	12 (24.5%)	6 (12.8%)	1 (33.3%)	Χ^2^=2.525	0.283
Furos	49 (49.5%)	25 (51.0%)	21 (44.7%)	3 (100%)	Χ^2^=3.543	0.170
Furos dose	70.98±56.3	80±70	57.27±36.2	93.3±23	Χ^2^=1.232	0.301
HCTZ	12 (12.1%)	4 (8.2%)	8 (17.0%)	0 (0%)	Χ^2^=2.194	0.334
Stat	92 (92.9%)	44 (89.8%)	45 (95.7%)	3 (100%)	Χ^2^=1.527	0.466
Allop	13 (13.1%)	8 (16.3%)	4 (8.5%)	1 (33.3%)	Χ^2^=2.392	0.302

Continuous variables: mean ± standard deviation; categorical variables: n (%). DD: deletion/deletion genotype; DI: deletion/insertion genotype; II: insertion/insertion genotype; T. diagn: time to disease diagnosis; BMI: body mass index; AC: abdominal circumference; SAH: systemic arterial hypertension; DM: diabetes mellitus; FH HF: family history of heart failure; FH CAD: family history of coronary artery disease; SBP1 and SBP2: systolic blood pressure at the first and second medical visits, respectively; DBP1 and DBP2: diastolic blood pressure at the first and second medical visits, respectively; HR1 and HR2: heart rate at the first and second medical visits, respectively; ΔSBP: difference between systolic blood pressure at the second and first medical visits; ΔDBP: difference between diastolic blood pressure at the second and first medical visits; ΔHR: difference between heart rate at the second and first medical visits; Hb: hemoglobin; UA: uric acid; TC: total cholesterol; Na: sodium; Cr: creatinine; CrCl: estimated creatinine clearance; QRS: QRS complex on electrocardiogram; LBBB: left bundle-branch block; BB: beta-blocker; ACEI: angiotensin converting-enzyme inhibitor; ARB: angiotensin receptor blocker; Spiro: spironolactone; Furos: furosemide; HCTZ: hydrochlorothiazide; Stat: statin; Allop: allopurinol; BB target: target dose of beta-blocker; ACEI target: target dose of angiotensin-converting-enzyme inhibitor; Furos dose: mean dose of furosemide at the last medical visit.

### Echocardiographic results

**Figure 1:**
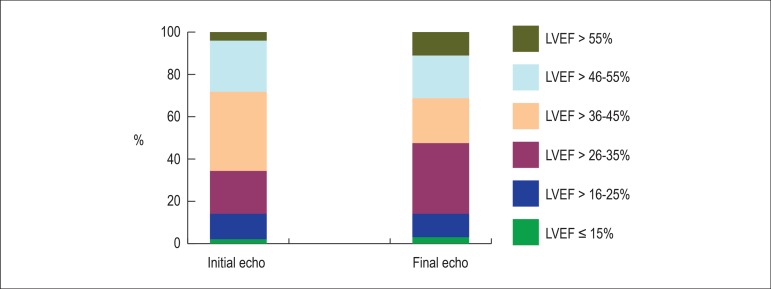
Echocardiographic findings of left ventricular ejection fraction (LVEF) at the initial and final tests in the study sample.

Figure 1 shows the LVEF findings at the initial and final echocardiographic tests.

Initially most patients (37.38%) were in the LVEF range of 35-45%, being followed by those in the LVEF range of 46-55% (24.24%). On the final echocardiogram, there was a change in that pattern, most patients (33.34%) being in the LVEF range of 26-35% (one LVEF range below that of most patients on the first test), followed by those in the LVEF range of 36-45% (21.21%) (one LVEF range below the second highest percentage of patients on the first echocardiogram).

Table 2 shows the mean values of LVEF, LVSD and LVDD on both echocardiograms assessed, without statistical difference between the values found.

**Table 2:** Echocardiographic parameters in the population and their evolution according to ACE gene polymorphisms (ACEGP)

Variable	Total ACEGP (n=99)	DD (n=49)	DI (n=47)	II (n=3)	F	p
LVEF1 (%)	38.84±11.11	39.51±9.39	38.50±12.36	33.33±18.90	0.475	0.623
LVSD1 (mm)	48.85±15.09	49.96±17.48	46.98±12.14	60±11.53	1.321	0.272
LVDD1 (mm)	62.21±15.71	63.31±20.37	60.57±8.99	70±9.64	0.739	0.480
LVEF2 (%)	38.45±13.71	36.07±14.29	40.83±12.87	40±14.80	1.487	0.231
LVSD2 (mm)	50.24±12.15	52.16±11.84	48.26±12.58	50±7	1.248	0.292
LVDD2 (mm)	62.39±10.03	63.71±10.29	61.23±9.98	59±2.65	0.909	0.407
ΔLVEF (%)	- 0.39 ±15.02	- 3.44±14.70	2.34±15.26	6.67±6.66	2.165	0.120
ΔLVSD (mm)	2.41±10.51	4.06±10.47	1.49±10.31	- 10 ± 4.60	2.991	0.055
ΔLVDD (mm)	1.46±8.79	2.98±8.94	0.68±8.12	- 11 ± 7.00	4.184	0.018

Variables expressed as mean ± standard deviation. ACEGP: angiotensin-converting-enzyme gene polymorphisms; DD: genotype deletion/deletion; DI: genotype deletion/insertion; II: genotype insertion/insertion; LVEF1: LV ejection fraction on the first echocardiogram; LVSD1: LV systolic diameter on the first echocardiogram; LVDD1: LV diastolic diameter on the first echocardiogram; LVEF2: LV ejection fraction on the final echocardiogram; LVSD2: LV systolic diameter on the final echocardiogram; LVDD2: LV diastolic diameter on the final echocardiogram; ΔLVEF: difference between LV ejection fraction on the final and on the first echocardiograms; ΔLVSD: difference between the LV systolic diameters on the final and on the first echocardiograms; ΔLVDD: difference between the LV diastolic diameters on the final and on the first echocardiograms; DD: deletion/deletion genotype; DI: deletion/insertion genotype; II: insertion/insertion genotype.

**Figure 2:**
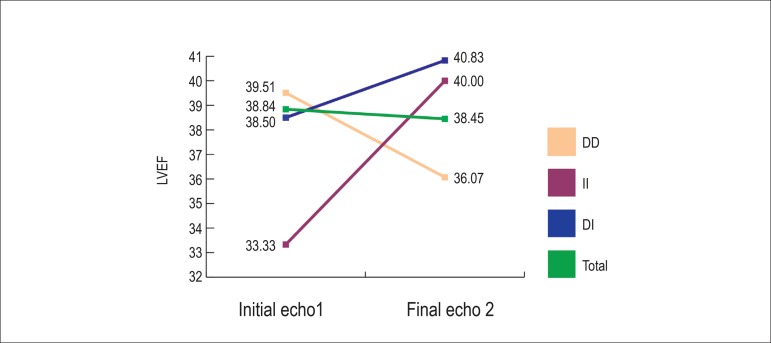
Mean left ventricular ejection fraction (LVEF) value changes during follow-up between final and initial echocardiographies in the sample and according to the genotype groups.

Figure 2 shows the mean LVEF value changes during follow-up between final and initial echocardiographies in the sample and in the genotype groups.

The changes during follow-up in the echocardiographic parameters, regarding their improvement or worsening, were objectively assessed, and the differences were quantified. Table 2 shows the differences between the two echocardiographic assessments of LVSD, LVDD and LVEF (ΔLVSD, ΔLVDD and ΔLVEF).

The ΔLVDD was positive in the sample and individuals with DD and DI genotypes, showing and increase in LVDD. Patients with II genotype had negative ΔLVDD (mean, -11), evidencing a reduction in LVDD. That ΔLVDD assessment was statistically significant in the analysis between the groups (p=0.018).

The ΔLVSD showed the same trend in the genotype groups and in the sample (increase in the DD and DI genotypes, and decrease in the II genotype), but with no statistical significance.

The ΔLVEF was negative in the sample and individuals with DD genotype, showing a decrease in LVEF, and positive in DI and II genotypes, showing an increase in LVEF. However, differently from ΔLVDD, there was no statistical significance.

To objectively assess whether there was improvement or worsening of the parameters analyzed (LVEF, LVDD and LVSD) during follow-up, FΔLVEF, FΔLVSD and FΔLVDD were obtained.

**Figure 3:**
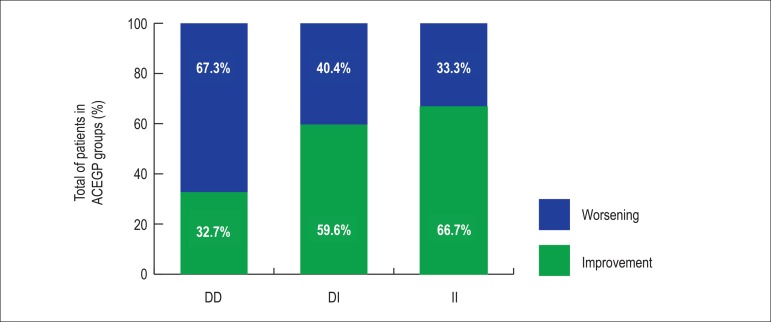
Left ventricular ejection fraction change during follow-up according to the genotype groups.

Figure 3 evidences, with statistical significance (Χ^2^= 7.497, p=0.024), the improvement or worsening during follow-up of LVEF (FΔLVEF) according to the ACEGP genotypes, with each cylinder representing 100.0% of genotype groups, and the colors green and blue representing the percentages of patients with LVEF improvement and worsening, respectively.

Worsening of the LVEF was observed in most DD genotype patients (67.3%), in 40.4% of DI genotype patients and in only 33.3% of genotype II patients.

Regarding FΔLVSD, worsening was observed in most DD genotype patients (65.4%) and in 40.4% of DI genotype patients, with statistical significance (p=0.010), while all II genotype patients (100.0%) had improvement of that parameter (Table 3).

**Table 3:** Analysis of left ventricular systolic diameter variation during follow-up (FΔLVSD) according to the genotype groups studied.

FΔLVSD	DD	DI	II	χ^2^	p
Improvement	17 (34.7)	28 (59.6)	3 (100.0)	9.233	0.010
Worsening	32 (65.3)	19 (40.4)	0 (0.0)		
Total	49 (100.0)	47 (100.0)	3 (100.0)		

DD: deletion/deletion genotype; DI: deletion/insertion genotype; II: insertion/insertion genotype; χ2: chi-square test.

**Figure 4:**
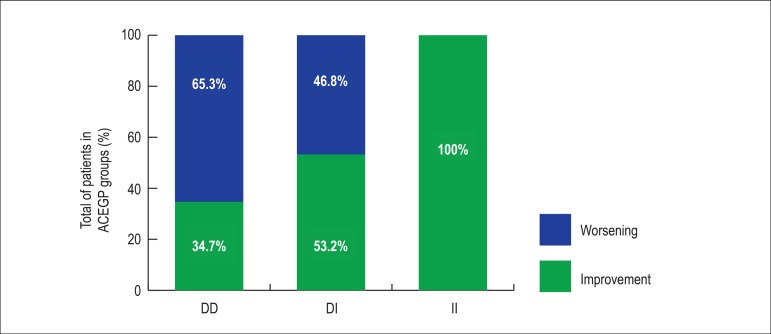
Left ventricular diastolic diameter variation during follow-up (FΔLVDD) according to the genotype groups studied.

The same analysis was performed for FΔLVDD, evidencing worsening, that is dilation, in 32 DD genotype patients (65.3%) and 22 (46,8%) DI genotype patients, but in no II genotype patient, with statistical significance (Χ^2^ = 7.023; p=0.030) (Figure 4).

Table 4 shows the correlations between the echocardiographic variables and D allele weight (Pearson correlation - r). Significant correlation was evidenced with ΔLVEF, ΔLVSD and ΔLVDD.

**Table 4:** Table of correlations with D allele weight

Variable	r	p
LVEF1	0.081	0.426
LVSD1	0.025	0.803
LVDD1	0.035	0.730
LVEF2	0.162	0.110
LVSD2	0.142	0.159
LVDD2	0.136	0.179
ΔLVEF	- 0.207	0.040
ΔLVSD	0.205	0.042
ΔLVDD	0.232	0.021

LVEF1: LV ejection fraction on the first echocardiogram ; LVSD1: LV systolic diameter on the first echocardiogram; LVDD1: LV diastolic diameter on the first echocardiogram; LVEF2: LV ejection fraction on the final echocardiogram; LVSD2: LV systolic diameter on the final echocardiogram; LVDD2: LV diastolic diameter on the final echocardiogram ΔLVEF: difference between LV ejection fraction on the final and on the first echocardiograms; ΔLVSD: difference between the LV systolic diameters on the final and on the first echocardiograms; ΔLVDD: difference between the LV diastolic diameters on the final and on the first echocardiograms.

## Discussion

The allele frequency obtained in this study differs from that of most national and international studies, because we found a lower number of II genotype patients, only 3% of the population. In patients with CAD or heart failure, higher D allele frequency than that in the general population and higher prevalence of the DI genotype have been reported, which differs from the findings in this study, where the DD genotype was the most prevalent.

Remodeling after acute myocardial infarction is a predictor of heart failure and mortality, and the increase in ventricular diameters in patients with heart failure is associated with clinical worsening. The renin-angiotensin-aldosterone system and ACE are known to contribute to those processes; thus, some studies assessing ACEGP in those populations have also assessed echocardiographic parameters, similarly to the present study. Higher serum levels of ACE and angiotensin II in patients with DD and DI genotypes can be related to worse outcome for those patients.

Some studies have reported different echocardiographic outcomes for patients with heart failure and CAD, depending on the ACEGP.^12^ Nagashima et al.^13^ have shown, in patients with old anteroseptal infarction, the higher influence of DD and DI genotypes on left ventricular remodeling as compared with that of II genotype patients. In addition, He et al.^14^ have reported that the I/D ACEGP can have an important role in late ventricular remodeling after acute myocardial infarction. Ohmichi et al.^9^ have shown that the D allele presence can be a risk factor for the development of heart failure with left ventricular dysfunction after acute myocardial infarction.

The present study found worsening in the LVEF ranges during follow-up, with most patients with ejection fraction values lower than those in the initial test, despite drug treatment. Analyzing the mean values of ejection fraction and ventricular systolic diameters, a trend towards worsening is observed in DD genotype individuals, but with no statistical significance between the ACEGP groups.

However, there was echocardiographic worsening of the mean values of LV diastolic volume in DD genotype patients, with an increase in the ΔLVDD, with statistical significance in the analysis between the ACEGP groups. In addition, the objective analysis of improvement or worsening of the echocardiographic parameters during follow-up evidenced, with statistical significance, more DD genotype patients with worsening, followed by DI genotype patients, while most II genotype patients improved those parameters. This suggests a pattern in which the D allele presence would be associated with worsening of echocardiographic parameters, more evident in the DD genotype group than in the DI genotype group.

Assessing the importance of the D allele, there was a significant correlation between its weight and the echocardiographic variables ΔLVEF, ΔLVSD and ΔLVDD, evidencing that, in that population, ACEGP associated with different echocardiographic outcomes, according to the D allele presence and genotypes of that polymorphism. Such results are in accordance with literature reports of higher severity of those patients and worse echocardiographic outcome.^5,12^

A Brazilian study conducted in 2005^15^ with patients with heart failure of all causes, 63 of ischemic etiology, has shown the trend towards greater left ventricular diameters, mainly the LVSD in patients with the DD genotype, meaning worse outcome for the DD genotype; however, the present study did not find the same statistical impact. Another study^16^ has reported the association of the D allele presence with left ventricular dysfunction in patients with acute myocardial infarction, but at a more acute phase of the infarction, differently from that proposed in the present study.

In a study^17^ assessing 142 patients with acute myocardial infarction, echocardiographic assessment including the measurement of LVEF and left ventricular diastolic and systolic volumes has shown no statistical difference between the mean values of the tests performed in each genotype group. However, differently from the results of the present study, those authors have reported improvement during follow-up of LVEF and both diameters in patients with the DD genotype, as well as improvement in LVEF during follow-up in DI genotype patients, but not in II genotype patients.

Other studies support the thesis that drug treatment with ACE inhibitors^18^ or with beta-blockers^12^ has a more positive influence on the echocardiographic parameters of DD genotype patients. The Russian study^18^ assessing patients with ischemic heart failure has reported greater improvement in ejection fraction and systolic and diastolic diameters in the DD genotype patients who started treatment with perindopril. In those studies, the rates of ACE inhibitor use were higher than those of this study, which, considering the reports on the benefit of the use of those drugs in D allele patients, could partially explain the difference in results; that, however, cannot be applied when analyzing the use of angiotensin-receptor blockers.^17^ The beta-blocker use across the genotypes was very high and very similar (100% DD, 95.7% DI and 100% II). In addition, the rates of use of ACE inhibitors or angiotensin-receptor blockers were high and similar, with no statistically significant difference in treatment according to the genotypes. The same occurs regarding the target dose of ACE inhibitors and beta-blockers, because, although higher doses could lead to a different outcome, the genotype groups studied did not significantly differ regarding the target dose.

Possible limitations of this study include the number of patients, mainly in the II genotype group; however, although several studies have larger samples,^19^ genetic studies with smaller numbers of patients have been reported.^20^ In addition, several pertinent results were obtained with evident statistical significance. The small number of patients with II genotype might somehow be related to the severity of the population studied, mostly composed by patients with DD and DI genotypes, reported as related to worse outcome. The analysis of genotype subgroups reported in the literature comprises always the three subgroups, without gathering any of them. This study evidenced the correlation of the D allele presence (none in II, one in DI and two in DD) with echocardiographic outcome, emphasizing the importance of analyzing each genotype separately, despite the difference in the number of patients in each genotype group. Another limitation relates to data collection from medical records, which can generate errors, but that was reduced by the fact that the population was cared for at a university center of teaching and research with experienced professionals.

## Conclusions

In a population of 99 patients with ischemic heart failure:

The allele and genotype frequencies related to ACEGP found in this study differed from those of the national and international literature. Only 3% of the population had II genotype.

The ACEGP studied associated with the echocardiographic outcome: there were more DD genotype patients with worsening of the LVEF, LVSD and LVDD, followed by DI genotype patients, while II genotype patients had the best outcome. Echocardiographic analysis of the difference between LVDD during follow-up showed the same pattern.
